# Supplementary material for: Photodynamic and Cold Atmospheric Plasma Combination Therapy Using Polymeric Nanoparticles for the Synergistic Treatment of Cervical Cancer
Source: Int J Mol Sci. 2021 Jan 25;22(3):1172. doi: 10.3390/ijms22031172 (PMC7865232; doi:10.3390/ijms22031172)
Supplement: Supplementary file 1 [file ijms-22-01172-s001.pdf]

Supplementary Information

Photodynamic and Cold Atmospheric Plasma Combination Therapy Using Polymeric Nanoparticles for the Synergistic Treatment of Cervical Cancer

Ji-Hui Ha and Young-Jin Kim*

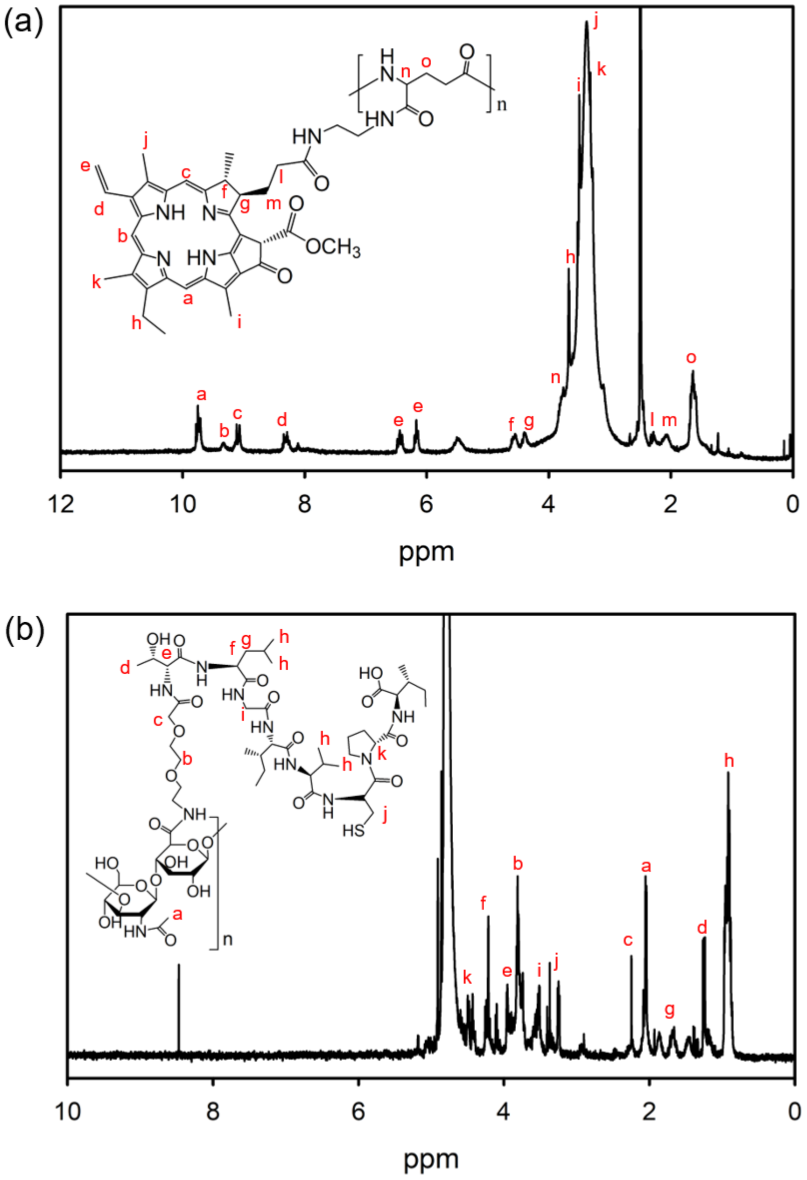

Figure S1. ^{1}H NMR spectra of (a) γ-PGA-Pheo a and (b) HA-EAE7.

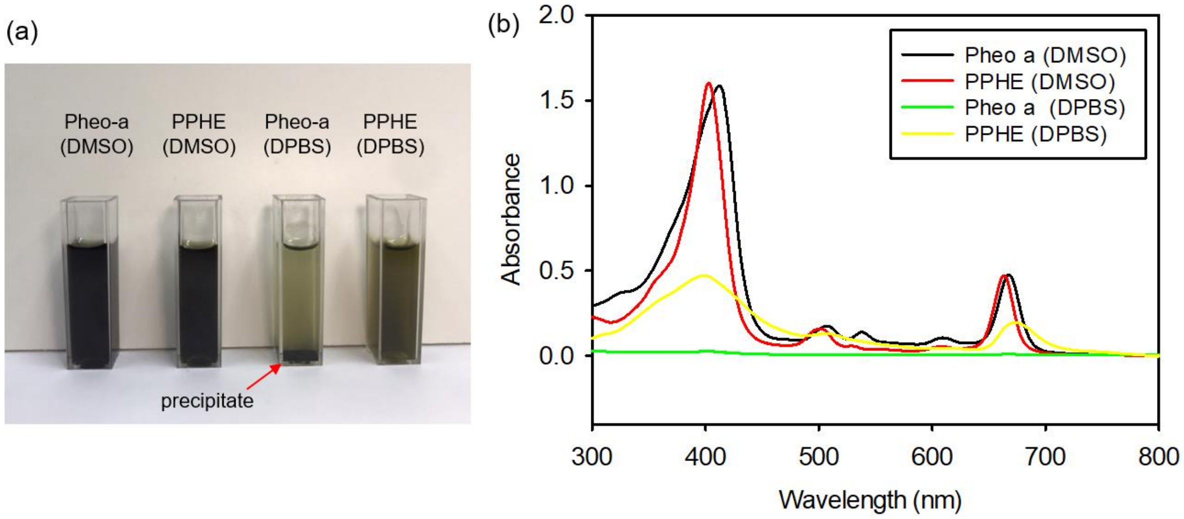

Figure S2. (a) Optical images of free Pheo a and PPHE dissolved in DMSO or DPBS (0.4 mg/mL Pheo a). The arrow shows the precipitate of Pheo a. (b) UV–visible spectra of free Pheo a and PPHE in DMSO or DPBS (10 μg/mL Pheo a).

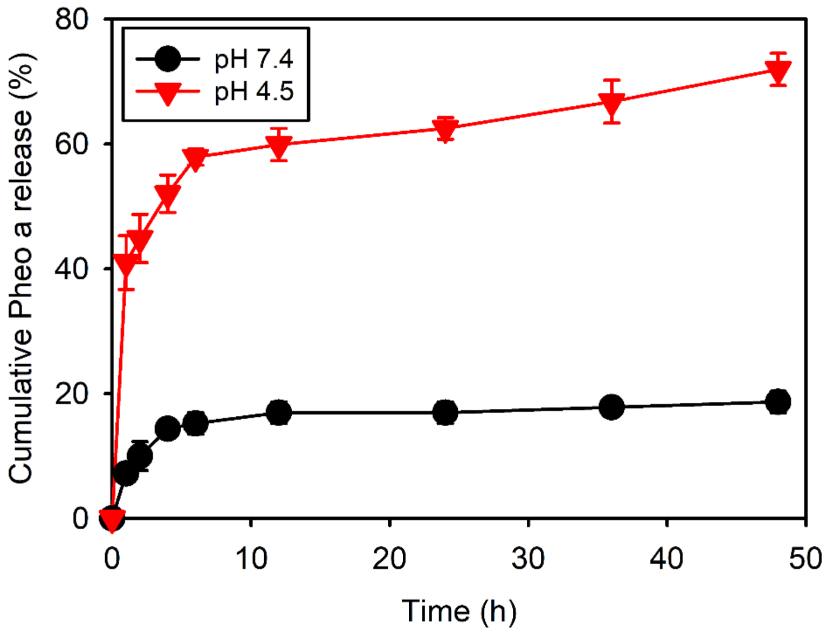

Figure S3. The cumulative release profiles of Pheo a from the PPHE polymeric nanoparticles in different pH conditions at 37 ℃ (*n* = 3).

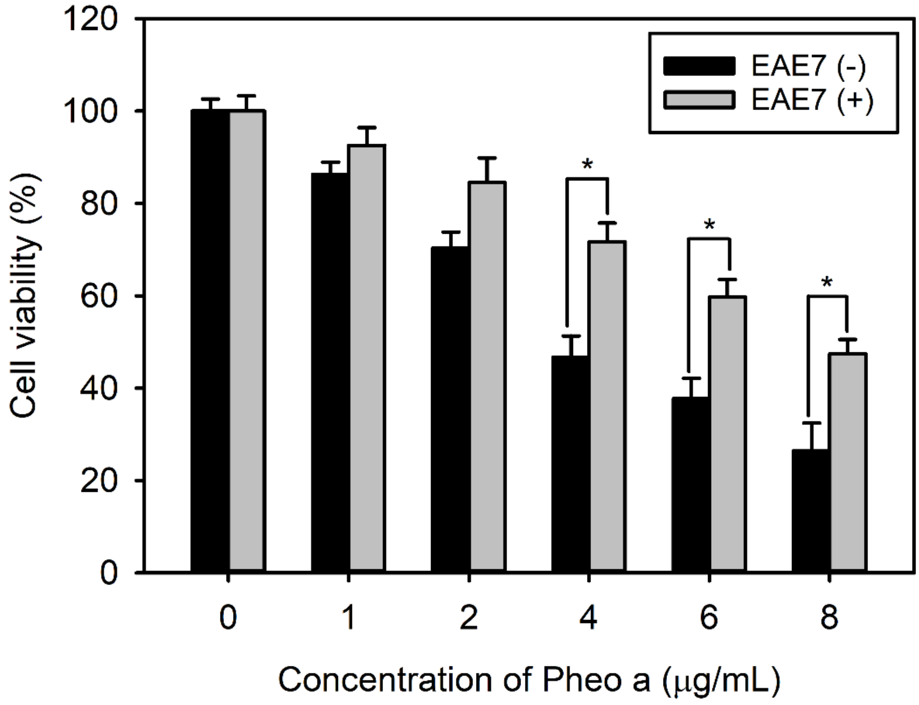

Figure S4. Phototoxicity of PPHE polymeric nanoparticles on CaSki cells after preincubation with (+) or without (−) excess free EAE7.

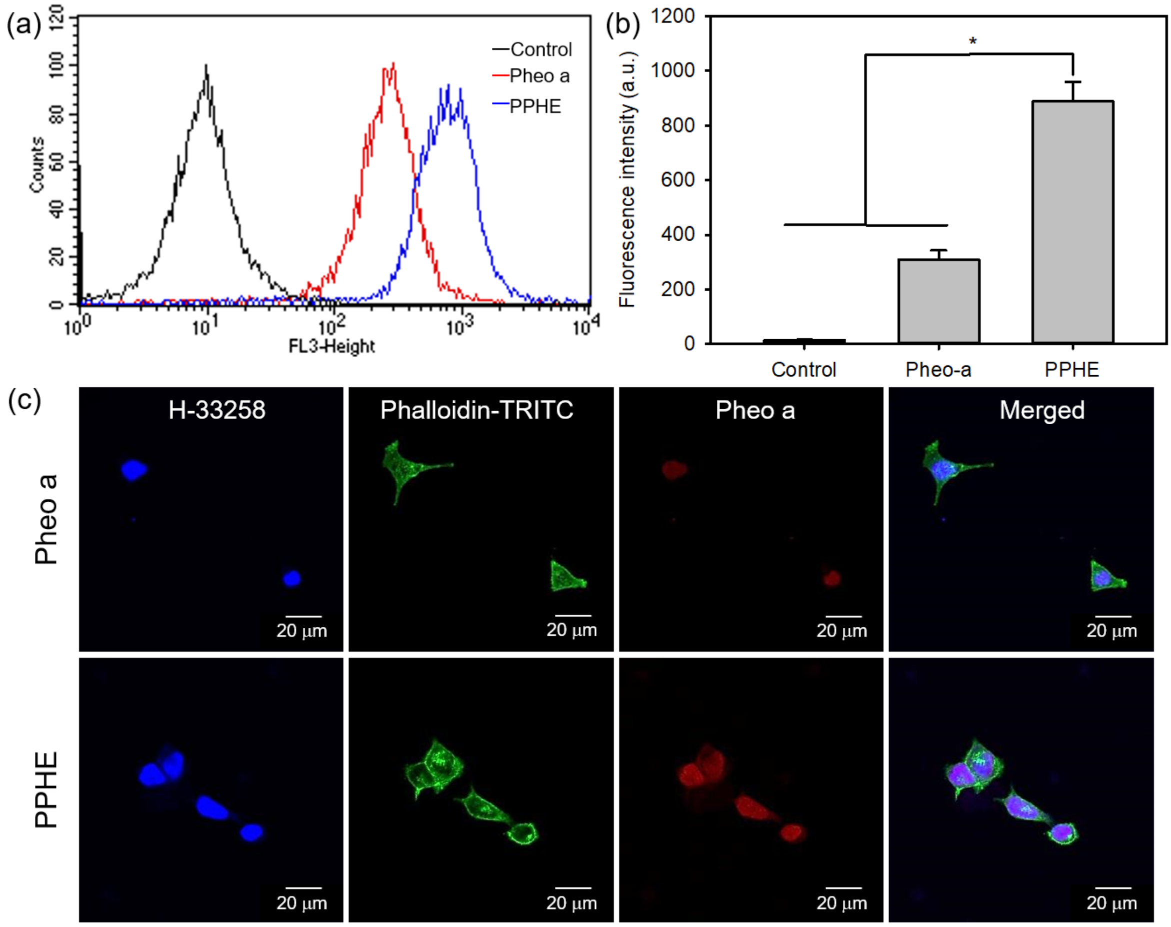

Figure S5. (a,b) Flow cytometric analysis of the intracellular uptake of free Pheo a and PPHE polymeric nanoparticles (4 μg/mL Pheo a) in CaSki cells after incubation for 2 h in the dark: (a) Shift in fluorescence peak and (b) fluorescence intensity due to the intracellular uptake of free Pheo a and PPHE polymeric nanoparticles (n = 5). (c) Confocal laser scanning microscopy images of the intracellular distribution of free Pheo a and PPHE polymeric nanoparticles (4 μg/mL Pheo a) in CaSki cells after incubation for 2 h in the dark.
